# RNA-seq Co-Expression Analysis Reveals a Midgut-Associated Digestive Gene Module in *Helicoverpa armigera*

**DOI:** 10.3390/biotech15030053

**Published:** 2026-07-13

**Authors:** Bairon J. Matabanchoy Pejendino, Vicente E. Mallama Cadena, María C. Díaz Rodríguez, Claudia Salazar Gonzalez, Pedro A. Velasquez-Vasconez

**Affiliations:** 1 Department of Production and Plant Protection, University of Nariño, Pasto 52001, Colombia; 2 School of Basic Sciences, Technology and Engineering, National Open and Distance University, Pasto 52001, Colombia

**Keywords:** serine proteases, polyphagous pests, plant-insect interactions, transcriptome, trophic plasticity

## Abstract

*Helicoverpa armigera* is one of the most destructive polyphagous pests, yet the transcriptional organization underlying its digestive capacity remains poorly resolved. Here, we compiled 579 publicly available RNA-seq libraries representing 54 independent experiments and quantified transcript abundance across tissues and developmental stages. This complete dataset was used to support broader tissue-level expression profiling. After metadata harmonization and quality filtering, a subset of 130 biologically comparable libraries from five tissue/developmental categories was retained for weighted gene co-expression network analysis. WGCNA identified four biologically informative modules, among which the turquoise module was positively associated with fourth- and fifth-instar larval midgut samples. Independent expression profiling revealed strong midgut-biased expression of several trypsin- and chymotrypsin-like serine proteases, although only a subset of these genes was assigned to the turquoise module. Descriptive functional annotation of this module identified 202 co-expressed loci, including digestive enzymes, nutrient transporters, detoxification-related proteins, epithelial components and putative transcriptional or signaling-associated genes. Phylogenetic analyses and manual inspection of genomic locations further showed that several digestive protease genes occur in local clusters and have closely related counterparts in *H. zea*, suggesting partial conservation of local genomic organization. Collectively, these results describe a midgut-associated co-expression module containing genes associated with digestive, absorptive and protective functions and provide candidate genes for future functional studies.

## 1. Introduction

*Helicoverpa armigera* (Hübner) (Lepidoptera: Noctuidae) is one of the world’s most damaging agricultural pests, and its success is tightly linked to midgut function. In particular, the cotton bollworm is a highly polyphagous moth of global significance, infesting a wide range of crops across Africa, Europe, Asia, and recently the Americas [1]. This pest’s larvae feed on hundreds of plant species and have evolved remarkable adaptations to overcome host plant defenses. For instance, *H. armigera* can tolerate diverse plant allelochemicals and defensive proteins, in part by deploying an extensive repertoire of detoxification enzymes and digestive proteases [1,2]. Notably, it compensates for plant protease inhibitors by overexpressing multiple serine protease genes, ensuring continued protein digestion even in inhibitor-rich diets [3,4]. Given the enormous economic losses attributed to this pest and its ability to thwart plant defense mechanisms, there is strong impetus to decipher the molecular biology of its midgut, the primary interface for food intake and defense confrontation.

Recent transcriptomic studies have begun to illuminate the complex biology of insect midguts, especially in Lepidoptera. Traditional gut transcriptomes have catalogued a multitude of digestion-related genes in caterpillars, including diverse proteases, carbohydrases, lipases, and nutrient transporters, as well as enzymes for detoxification of plant toxins [5,6]. These findings underscore how larvae like *H. armigera* can efficiently break down various plant tissues and neutralize chemical defenses. Midgut-focused studies have also identified immune and protective factors. For example, components of the peritrophic membrane and antimicrobial proteins reflect the midgut’s role in pathogen defense [7]. Despite these advances in profiling midgut gene expression, our understanding of the co-expression relationships among candidate genes involved in midgut function remains limited. Most insect gut transcriptomes to date have been descriptive or focused on differential expression under specific conditions (e.g., infection, toxin exposure), without reconstructing the underlying gene co-expression relationships [8]. In Lepidoptera, in particular, we lack a comprehensive view of how digestive and metabolic genes show coordinated expression patterns across midgut-related datasets.

Systems-level approaches such as gene co-expression network analysis offer a powerful means to identify functional gene modules in complex transcriptomes. By grouping genes with correlated expression patterns, weighted gene co-expression network analysis (WGCNA) can reveal sets of genes with correlated expression patterns that may participate in related biological processes. This approach has been successfully applied in insect studies to discover co-expressed gene modules and their co-expression modules. For example, identifying key hormonal regulators in a moth’s developmental network [9] or modules linked to physiological traits in other pests [10]. Coupling co-expression analysis with comparative genomics further enhances the discovery of critical modules. Conserved gene clusters across species may point to fundamental digestive processes, whereas lineage-specific expansions can highlight unique adaptations in polyphagous insects [1]. In the context of *H. armigera*, such integrated analysis show correlated expression patterns across the selected tissues. Moreover, identifying co-expression links between transcription factors or signaling genes and effector genes (like proteases or transporters) would provide candidate genes and hypotheses for future studies of midgut physiology.

One outstanding question in Lepidopteran biology is the functional significance of the many duplicated digestive enzyme genes, particularly proteases, found in these insects. Some researchers hypothesize that these gene expansions confer functional diversification, enabling different proteases to target various substrates or operate under different dietary conditions, and thereby enhance the insect’s ability to exploit diverse hosts. Other evidence, however, points toward a high degree of redundancy among protease genes, suggesting that extra copies primarily provide backup capacity to maintain digestion when some enzymes are inhibited or lost [2,11]. For example, knocking out a large cluster of trypsin genes in *H. armigera* induces compensatory upregulation of other protease genes, resulting in little net loss of total digestive activity [2]. This debate highlights the need for network-level analyses: if duplicated protease genes consistently appear in the same co-expression module or are co-expressed with specific inhibitor-resistant factors, it would support the redundancy/compensation model, whereas distinct modules might imply specialized roles. Clarifying these relationships is important for understanding how pests fine-tune their digestive machinery in response to plant defenses.

In this study, we address these gaps by compiling and harmonizing publicly available RNA-seq libraries for *H. armigera*, with an emphasis on tissue-associated gene co-expression. Using a subset of biologically comparable samples, we applied WGCNA to identify modules of genes with correlated expression patterns across selected tissues and developmental stages. Our goals were to identify midgut-associated co-expression modules, describe the annotated composition of the genes assigned to these modules, and prioritize digestive and transcriptional or signaling-associated candidates for future functional studies. Through comparative analysis, we also examined how selected serine protease genes relate to known digestive gene families and local genomic organization in *H. armigera* and *H. zea*. Notably, we identified a midgut-associated co-expression module that contains digestive serine proteases together with genes associated with transport, epithelial function, detoxification, transcription and signaling. Overall, our findings provide a descriptive co-expression framework for investigating larval midgut function and candidate genes relevant to digestive adaptation in this polyphagous pest.

## 2. Materials and Methods

### 2.1. RNA-seq Data Retrieval

Publicly available *Helicoverpa armigera* transcriptomes were downloaded from the NCBI Sequence Read Archive (SRA) with the SRA-Toolkit v3.2.1 [12]. A total of 579 publicly available RNA-seq libraries were retrieved. After quality control, metadata harmonization and selection of biologically comparable tissues, a subset of libraries representing whole-body early instars, fourth-instar midgut, fifth-instar midgut, pupal fat body and adult antennae was retained for WGCNA. Of the 579 RNA-seq libraries retrieved, 130 libraries representing five harmonized biological categories were retained for WGCNA: whole-body early instars (*n* = 17), fourth-instar midgut (*n* = 13), fifth-instar midgut (*n* = 57), pupal fat body (*n* = 28) and adult antennae (*n* = 15). To reduce heterogeneity in the WGCNA dataset, only biological categories represented by at least 12 RNA-seq libraries and supported by consistent metadata were retained. Categories with lower representation, ambiguous tissue assignment, specific experimental treatments, or narrowly defined developmental conditions were excluded from WGCNA. This filtering strategy was used to retain categories with sufficient replication and to improve biological comparability among samples used for network construction.

The corresponding accession numbers and biological metadata are available in Figshare: https://doi.org/10.6084/m9.figshare.32381694 (accessed on 6 July 2026).

### 2.2. Quality Control and Preprocessing

Raw reads were inspected with FastQC v0.12 to assess per-base quality, GC content and adaptor contamination [13]. Illumina adaptors and low-quality bases (Phred < 30) were removed with Trimmomatic v0.39 using parameters ILLUMINACLIP:2:30:10 LEADING:3 TRAILING:3 SLIDINGWINDOW:4:30 MINLEN:36 [14]. Only trimmed pairs that survived in both mates were retained for downstream analysis.

### 2.3. Transcript Quantification

Filtered reads were pseudo-aligned to the *H. armigera* reference transcriptome (GCF_030705265.1) with Kallisto v0.50.1 using default k-mer and bootstrap settings [15]. Transcript abundances were exported as transcripts-per-million (TPM) and summarised to gene level in R v4.4 [16].

### 2.4. Weighted Gene Co-Expression Network Analysis (WGCNA)

Log-transformed TPM-derived gene-level expression values were used to construct expression matrices for WGCNA using the WGCNA package v1.73 [17]. Before network construction, the gene-level expression matrix was filtered to remove genes with missing values and genes with zero total expression across the retained samples. Samples with zero total expression, if present, were also removed. A variance-stabilizing transformation was then applied, and genes were further filtered by variance. Specifically, only genes with variance above the 95th percentile were retained for network construction, resulting in 822 genes included in the final WGCNA matrix.

The suitability of the resulting matrix was evaluated using goodSamplesGenes, pre-WGCNA hierarchical sample clustering and objective outlier detection based on standardized sample connectivity (Z.k). No evident outlier samples were detected using the Z.k < −2.5 threshold. Soft-thresholding powers from 1 to 20 were evaluated using the scale-free topology fit index and mean connectivity. A soft-thresholding power of 14 was selected as a compromise between approximation to scale-free topology and preservation of network connectivity.

The final network was constructed as a signed network using biweight midcorrelation, signed topological overlap, a minimum module size of 20, deepSplit = 4 and mergeCutHeight = 0.15. Four co-expression modules were retained, while genes not confidently assigned to a coherent module were retained in the grey category. Module–trait relationships and eigengene heatmaps were visualized with ggplot2 [18]. Module membership, gene significance and intramodular connectivity were calculated to support descriptive candidate prioritization within modules. The R workflow used for WGCNA preprocessing, network construction and module analysis is available in Zenodo at https://doi.org/10.5281/zenodo.21046330 (accessed on 6 July 2026).

### 2.5. Identification of Serine Protease Genes

Predicted proteins were inspected for trypsin/chymotrypsin-like serine protease domains (IPR001254). Candidate proteins were further inspected for the presence and conservation of the catalytic triad residues His57, Asp102 and Ser195, according to the chymotrypsin numbering system. Proteins lacking one or more catalytic residues were classified separately as serine protease homologs or putatively inactive homologs rather than as active digestive proteases. Substrate-specificity classification was based on inspection of the substrate-binding pocket, particularly the residue corresponding to position 189 in the chymotrypsin numbering system. Proteins with Asp189 were classified as trypsin-like, whereas proteins with non-Asp residues at this position were classified more cautiously as chymotrypsin-like or other S1-family serine protease-like candidates, depending on domain architecture and conservation of catalytic residues.

### 2.6. Tissue-Specific Expression Profiling

TPM values of the curated serine proteases and their endogenous inhibitors were averaged by tissue; mean ± SD were plotted as bar charts and heat-maps with *ggplot2* [18]. For tissue-specific expression profiling of curated serine proteases, we used the broader 579-library dataset, because this analysis aimed to describe expression patterns across all available tissues and developmental stages. In contrast, WGCNA was restricted to the harmonized subset of 130 biologically comparable libraries.

### 2.7. Phylogenetic Analysis

Protein sequences of the curated trypsins and chymotrypsins were aligned with MAFFT v7 using the L-INS-i strategy [19]. The best-fit substitution model (LG + G4) was selected within IQ-TREE v2.3.5 [20]. Node support was estimated with 1000 bootstrap replicates. The tree was annotated in FigTree v1.4.4 [21]. To examine the local genomic organization of selected protease genes, the chromosomal locations of closely related *H. armigera* paralogs and their putative *H. zea* orthologs were manually inspected in the NCBI genome annotation records. Genes were considered locally clustered when they occurred in close physical proximity within the same chromosome or genomic scaffold. This inspection was used to determine whether selected trypsin- or chymotrypsin-like genes showed conserved local proximity between both *Helicoverpa* species; therefore, these observations represent descriptive evidence of conserved local genomic organization rather than a formal genome-wide microsynteny analysis.

## 3. Results

### 3.1. Large-Scale Co-Expression Analysis Delineates an Midgut-Associated Module

**Figure 1:**
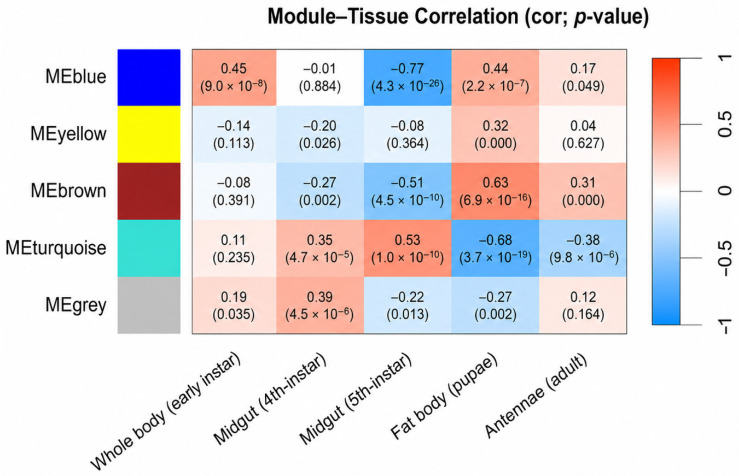
Correlation between module eigengenes and tissue or developmental-stage traits. Heatmap showing Pearson correlations between module eigengenes identified by WGCNA and five biological sample categories: whole-body early instar (n = 17), fourth-instar midgut (n = 13), fifth-instar midgut (n = 57), pupal fat body (n = 28) and adult antennae (n = 15). Each cell displays the correlation coefficient and its associated *p*-value in parentheses; red indicates positive correlations and blue indicates negative correlations. The grey category represents genes not assigned to a defined co-expression module.

**Figure 2:**
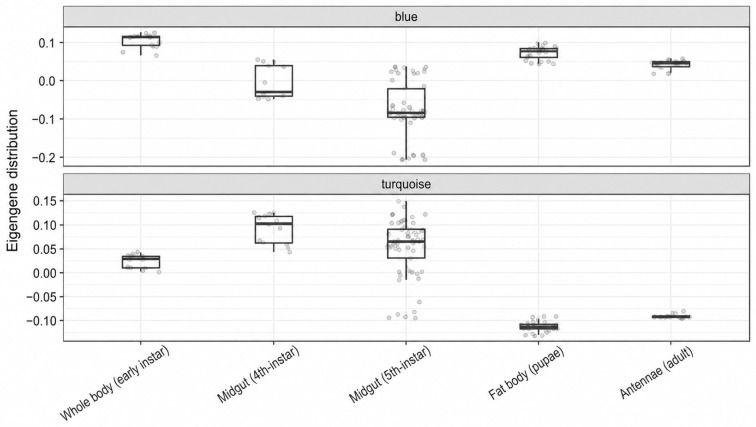
Distribution of module eigengene expression across tissue and developmental-stage categories for the blue and turquoise co-expression modules. Boxplots represent the distribution of eigengene values for the blue and turquoise modules across whole-body early-instar, fourth-instar midgut, fifth-instar midgut, pupal fat body and adult antenna samples; individual points correspond to individual samples.

Processing SRA runs yielded 579 TPM matrices representing 54 independent experiments covering larval, pupal and adult samples. A signed weighted gene co-expression network was constructed using a soft-thresholding power that achieved an approximate scale-free topology fit of $R^{2}\geq 0.90$ (Supplementary Figure S1). Hierarchical clustering and standardized connectivity analysis did not identify evident outlier samples (Supplementary Figures S2 and S3). The analysis identified four biologically informative co-expression modules including: blue, yellow, brown and turquoise, while genes not confidently assigned to a coherent module were retained in the grey category (Supplementary Figure S4). Module–tissue correlation analysis showed that the turquoise module was positively associated with larval midgut tissues, particularly fifth-instar midgut samples (r = 0.53, *p* = 1.0 × 10^−10^) and fourth-instar midgut samples (r = 0.35, *p* = 4.7 × 10^−5^). Conversely, this module was negatively associated with pupal fat body (r = −0.68, *p* = 3.7 × 10^−19^) and adult antennae (r = −0.38, *p* = 9.8 × 10^−6^), supporting its interpretation as a larval midgut-associated co-expression module. In contrast, the brown module was positively associated with pupal fat body (r = 0.63, *p* = 5.9 × 10^−16^) and negatively associated with fifth-instar midgut (r = −0.51, *p* = 4.5 × 10^−10^), whereas the blue module showed its strongest negative association with fifth-instar midgut (r = −0.77, *p* = 4.3 × 10^−26^) (Figure 1). The eigengene distribution analysis further confirmed these contrasting tissue-associated patterns: the turquoise module exhibited higher eigengene values in fourth- and fifth-instar midgut samples, but markedly lower values in pupal fat body and adult antennae (Figure 2). Conversely, the blue module showed reduced eigengene values in midgut samples, especially in fifth-instar midgut, while displaying higher values in whole-body early-instar, pupal fat-body and adult antenna samples (Figure 2).

### 3.2. Midgut-Biased Serine Proteases Dominate the Digestive Transcriptome

**Figure 3:**
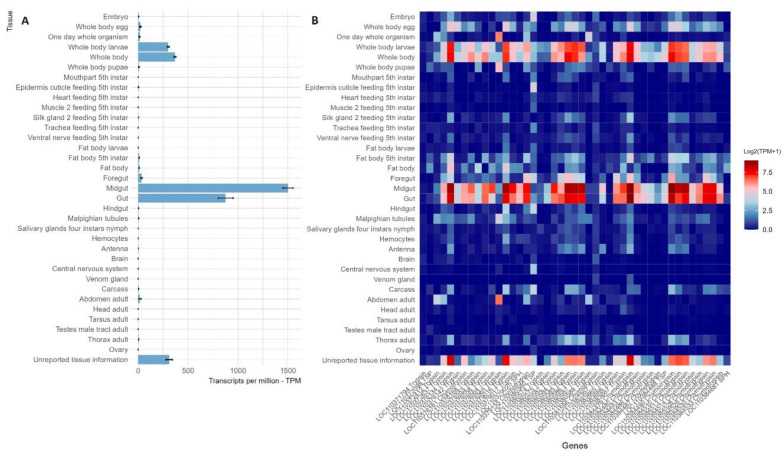
Expression profiles of serine protease genes in *Helicoverpa armigera* across tissues and developmental stages. (**A**) Mean expression levels of TPM of the selected set of serine protease genes across 36 tissues and developmental stages. (**B**) Heatmap showing the log-transformed expression (TPM) of individual serine protease genes across the same tissues.

Mean transcript abundance of serine-protease genes exceeded 1500 TPM in larval midgut and ~800 TPM in whole-gut samples, while early whole-body libraries averaged >250 TPM (Figure 3A). Five trypsins and four chymotrypsins reached >7.5 Log2(TPM + 1) and were almost exclusively expressed in the midgut (Figure 3B). A single trypsin gene, *LOC110378565*, displayed low midgut expression but high abundance in adult abdomen, pupal tissue and first-instar larvae (Figure 3B).

### 3.3. Phylogenetic Relationships and Local Genomic Organization of Protease Genes

Closely related trypsin and chymotrypsin paralogs clustered together by both expression and sequence similarity (Supplementary Figures S5 and S6). For instance, *LOC110380585* and *LOC110380587*, both annotated as trypsin-like serine proteases, lie 3 kb apart on chromosome 3 and share nearly identical midgut-biased profiles; their *H. zea* orthologs are likewise tandem on chromosome 1. Numerous linked protease pairs suggesting partial conservation of local genomic organization, which may be relevant to the shared expression patterns observed among selected digestive protease genes.

### 3.4. Expression Divergence Among Paralogs and Orthologs

Although trypsin paralogs *LOC110383667* and *LOC110378558* are separated by only 15 kb on chromosome 3, the former reached 7.5 Log2(TPM + 1) in midgut, whereas the latter peaked at 6.8 Log2(TPM + 1) (Figure 3B). Cross-species comparison revealed that the *H. armigera* gene *LOC110380582* (trypsin, chromosome 3, 7.5 Log2(TPM + 1)) corresponds to *LOC124635462* on *H. zea* (trypsin, chromosome 1), underscoring lineage-specific genomic rearrangements (Figure 3B). For chymotrypsins, the orthologous pair *LOC110384483* (*H. armigera*) and *LOC124636178* (*H. zea*) showed low midgut expression (4.7 Log2(TPM + 1)), whereas paralog *LOC110384490* was highly expressed (7.5 Log2(TPM + 1)) (Figure 3B).

### 3.5. Descriptive Annotated Composition of the Midgut-Associated Co-Expression Module

Descriptive functional annotation of the midgut-associated turquoise module identified encoded products for 202 co-expressed loci (Supplementary Table S1). Because a formal enrichment analysis was not performed, these annotations are interpreted as a qualitative description of the module composition rather than as statistically supported overrepresentation of specific biological processes. The module included genes associated with digestive, transport, detoxification, epithelial and signaling-related functions. Digestive components included three trypsin genes (*LOC110376142*, *LOC110378553* and *LOC110384045*), one chymotrypsin gene (*LOC110375658*), a serine protease-like gene (*LOC110381726*), three carboxypeptidase-related genes (*LOC110372081*, *LOC110373545* and *LOC126054783*), and genes encoding lipid- and carbohydrate-processing enzymes, including bile salt-activated lipase, pancreatic lipase-related protein 2 and lactase/phlorizin hydrolase. The module also contained multiple nutrient and solute transporters, including trehalose, amino acid, organic cation and inorganic phosphate transporters, consistent with functions related to nutrient digestion and absorption. In addition, several genes annotated as transcriptional and signaling-associated components were identified, including a GATA-binding factor (*LOC110374834*), a fork head domain transcription factor (*LOC110383619*), a POU domain protein (*LOC110379817*), a serine-threonine kinase receptor-associated protein (*LOC110377186*) and a 5-hydroxytryptamine receptor (*LOC110380291*). Detoxification- and epithelial-function-related genes, such as cytochrome P450 6B5, glutathione S-transferase 1, UDP-glycosyltransferase UGT5 and mucin-17, further indicate that the turquoise module contains genes associated with digestion, nutrient transport, signaling and protection against dietary xenobiotics.

## 4. Discussion

### 4.1. Digestive Enzymes and Adaptation

The larval midgut of *H. armigera* is a major site of enzyme production, reflecting evolutionary adaptations for efficient protein digestion. We identified a striking overexpression of serine proteases, particularly trypsins and chymotrypsins, in the midgut, underscoring their central role in breaking down dietary proteins. This indicates that a network of co-expressed genes underlies the specialized function of the larval midgut in protein digestion. This enzymatic arsenal is thought to underlie *H. armigera*’s extreme polyphagy, enabling it to exploit diverse host plants by overcoming plant defense proteins. Indeed, generalist herbivores often possess expanded multigene families of digestive proteases as an adaptive strategy [22]. *H. armigera* harbors dozens of trypsin- and chymotrypsin-like genes in its genome with significant sequence variability, especially in regions interacting with protease inhibitors. Recent evidence also indicates that plant defensive proteins can induce broad metabolic responses in *H. armigera* larvae [23]. Similar findings have been reported in other insects; for example, WGCNA in whitefly identified a module containing several midgut-expressed genes, including digestive proteases and detoxification enzymes [24]. Likewise, co-expression analysis in diamondback moth larvae highlighted sets of genes (e.g., aminopeptidases, alkaline phosphatase, cadherin, trypsin) that co-vary in expression and contribute to Bt toxin resistance, underscoring how functional modules can be tied to midgut physiology [25]. Together, these network studies demonstrate that midgut-associated gene modules are a common feature, grouping digestive enzymes with other co-expressed genes relevant to feeding and metabolism.

### 4.2. High Expression of Trypsin and Chymotrypsin Genes in the Generalist Insect Midgut

*H. armigera*, a polyphagous lepidopteran pest, exhibits exceptionally high expression of multiple trypsin- and chymotrypsin-coding genes in the larval midgut. Transcriptomic analyses confirm that many of these serine protease genes are midgut-biased, showing much higher expression in gut tissue compared to other tissues [26]. This aligns with the midgut’s role as the primary site of protein digestion under the highly alkaline conditions characteristic of lepidopteran larvae [26]. Notably, both *H. armigera* and its close relative *H. zea* share a conserved set of midgut serine proteases that are orthologous between the species and abundantly expressed, reflecting evolutionary conservation of digestive function [27]. However, comparative genomics also reveals divergence. *H. armigera* possesses a larger repertoire of these enzymes than *H. zea*, owing to gene expansion in the *Helicoverpa* lineage. For instance, *H. armigera* has 29 clade-1 trypsin genes (28 of which occur in one genomic cluster) and 26 clade-1 chymotrypsin genes, whereas *H. zea* has somewhat fewer due to gene loss or incomplete assemblies [27]. Despite this difference in gene copy number, many orthologous trypsin/chymotrypsin genes in *H. armigera* and *H. zea* remain highly expressed in the midgut of both species, indicating evolutionary constraint on their digestive roles even as the gene family diversified. These midgut-biased proteases often rank among the most highly expressed transcripts in larval guts, emphasizing their critical contribution to nutrient acquisition and generalist feeding habits.

### 4.3. Gene Duplication, Divergence, and Digestive Enzyme Evolution

The expansion of trypsin and chymotrypsin gene families is a hallmark of generalist herbivorous insects and has important evolutionary implications. Multiple studies have linked gene family amplification to dietary breadth and adaptation. In Lepidoptera, large arrays of serine protease genes (many arising from gene duplications) are thought to confer a fitness advantage by providing “backup” enzymes and enabling functional diversification—an insect with many protease isoforms can better cope with various protein sources and plant defensive inhibitors. For example, *Spodoptera frugiperda* (fall armyworm, a polyphagous pest) carries at least 14 chymotrypsin and 9 trypsin genes, whereas a more specialist lepidopteran like *Diatraea saccharalis* (sugarcane borer) has only ~9 and 4, respectively [28]. *H. armigera* similarly shows an enlarged set of digestive serine proteases, with many trypsin/chymotrypsin genes identified in its genome.

Many of these arose via tandem duplications in the genome, forming clusters of related paralogs. This pattern of gene clustering has been observed in insects such as *Drosophila melanogaster*, *Anopheles gambiae*, and *Manduca sexta*, where tandem duplications and unequal crossing over events have played crucial roles in expanding these gene families [29,30,31]. These processes contribute to the generation of related paralogous clusters, which may contribute to functional diversification within serine protease genes. *H. armigera’s* large trypsin and chymotrypsin clades, amino acid identities between paralogs can be as low as ~45–50%, reflecting neofunctionalization or subfunctionalization [27]. Some duplicated proteases maintain the canonical catalytic triad and substrate-binding residues, preserving proteolytic function, while others have mutated critical residues (serine protease homologs), potentially serving as protease inhibitors or decoys [28].

This diversification likely allows generalist insects to broaden their dietary range; they can express different protease variants to efficiently digest diverse plant proteins or to overcome a variety of plant protease inhibitors. Indeed, experimental adaptation of *H. armigera* to diets containing soybean protease inhibitors triggers differential upregulation of a subset of trypsin/chymotrypsin genes. In one study, eight serine peptidase genes, including an unusual “polytrypsin” (containing two trypsin domains) were strongly induced in PI-fed larvae, presumably compensating for inhibitor-blocked enzymes [4]. Such findings support the idea of dosage and diversity selection: having many protease genes allows insects to adjust gene expression dosage and employ variant proteases with altered inhibitor sensitivity or substrate range [32,33,34]. Over evolutionary time, this has enabled generalist Lepidoptera like *Helicoverpa* and *Spodoptera* to thrive on a wide array of host plants.

### 4.4. Co-Expressed Transcriptional and Signaling-Associated Genes

A notable feature of the midgut-associated co-expression module in *H. armigera* is that it contains not only digestive enzymes but also genes annotated as transcriptional or signaling-associated components, including transcription factors, kinases and receptor-like proteins (Supplementary Table S1). The tight co-expression of these genes with digestive enzymes suggests that these genes share expression patterns with digestive enzymes in the retained WGCNA dataset. Generalist Lepidoptera genomes contain large clusters of trypsin and chymotrypsin genes with previously reported regulatory features, leading to highly conserved, correlated expression patterns in the larval midgut [27]. Recent insect comparative-genomic studies have also used chromosome-level assemblies and transcriptomic evidence to connect gene-family organization with metabolic, defensive or life-history adaptation [35,36]. Functional studies in *H. armigera* show that even when entire clusters of up to 18 trypsin genes were deleted, compensatory upregulation of other proteases ensured digestive stability, underscoring the robustness of digestive gene expression responses [2].

Previous studies have associated several transcription factor families with gut-specific gene expression in insects. GATA family transcription factors have emerged as crucial regulators of insect midgut genes. *H. armigera* and *S. frugiperda* encode multiple GATA factors, one of which is predominantly expressed in the larval midgut and can activate the promoters of midgut-expressed genes [37]. The HaGATAe gene has been shown to bind to GATA-like promoter elements in the regulatory regions of key midgut-associated genes (like Bt toxin receptors and alkaline phosphatase), enhancing their transcription [38]. These studies provide biological context for the presence of GATA-related genes in midgut-associated datasets, but the present analysis does not establish direct regulatory relationships between GATA factors and serine protease genes.

Several signaling-associated genes were also identified within the turquoise module. Previous studies have linked nutritional and hormonal signaling pathways with physiology in insects [39]. Although these observations provide useful biological context, the present co-expression analysis does not allow direct inference of signaling pathways or regulatory interactions. Therefore, these genes should be regarded as candidates for future functional studies (Supplementary Table S1).

Previous studies provide useful biological context for interpreting the presence of transcriptional and signaling-associated genes in the midgut-associated module. Nutritional, hormonal and transcriptional pathways have been linked to digestive physiology in insects, including TOR-related feeding responses, ecdysone-dependent regulation of digestive enzymes and transcriptional cofactors associated with diet-responsive gene expression [39,40,41,42]. However, the present study did not directly test these pathways or regulatory mechanisms. Therefore, these comparisons should be interpreted as hypotheses for future functional work rather than as conclusions supported by the current co-expression analysis.

Overall, the identification of such co-expression modules—containing proteases, amino acid transporters, intestinal peptides, and transcription factors—supports their prioritization as candidates for future functional validation, without implying direct regulatory control. These co-expressed genes should be interpreted as candidates for future functional validation rather than as confirmed regulators of digestive enzyme expression.

### 4.5. This Study Has Several Limitations

First, the analysis integrates publicly available RNA-seq libraries generated across independent experiments, potentially introducing technical and biological heterogeneity. Co-expression relationships do not demonstrate direct regulatory interactions and should therefore be validated experimentally. Although this module is referred to here as midgut-associated, it should not be interpreted as originating from a single cellular population. The present analysis was based on bulk RNA-seq datasets and did not include single-cell RNA-seq libraries; therefore, formal assignment of module genes to individual midgut cell types was not possible. Indeed, because a formal enrichment analysis was not performed, annotated composition of the turquoise module should be interpreted as a descriptive annotation rather than as evidence of statistically significant enrichment or functional specialization. Functional annotations depend on the completeness and accuracy of the available genome annotation. Nevertheless, the identified midgut-associated module provides a prioritised set of digestive and transcriptional or signaling-associated candidate genes for future functional studies.

## 5. Conclusions

This integrative analysis of 579 publicly available RNA-seq libraries provides a broad tissue-level view of gene expression in *H. armigera*. Using a harmonized subset of 130 biologically comparable libraries, WGCNA identified a co-expression module strongly associated with the larval midgut. The turquoise module comprised 202 annotated co-expressed loci, including digestive enzymes, nutrient transporters, detoxification-related proteins, epithelial components and transcriptional or signaling-associated genes. In parallel, several trypsin- and chymotrypsin-like genes displayed strong midgut-biased expression, highlighting the central role of serine proteases in larval digestive physiology. Phylogenetic relationships and manual inspection of genomic locations further indicated that selected protease genes occur in local clusters and have closely related counterparts in *H. zea*. Although functional validation is required, the genes prioritised in this study provide a useful foundation for investigating digestive plasticity and for guiding future functional screening of candidate genes potentially relevant to pest management.

## Data Availability

The dataset supporting the findings of this study is publicly available in Figshare at: https://doi.org/10.6084/m9.figshare.32381694 (accessed on 6 July 2026). The dataset contains the accession identifiers and associated metadata of the publicly available *Helicoverpa armigera* RNA-seq libraries retrieved from the NCBI Sequence Read Archive (SRA) and used for the transcriptomic and gene co-expression analyses performed in this study. The R workflow used for WGCNA preprocessing, network construction and module analysis is publicly available in Zenodo at https://doi.org/10.5281/zenodo.21046330 (accessed on 6 July 2026).

## Supplementary Materials

The following supporting information can be downloaded at: https://www.mdpi.com/article/10.3390/biotech15030053/s1. Figure S1: Selection of the soft-thresholding power for weighted gene co-expression network analysis (WGCNA); Figure S2: Objective detection of outlier samples prior to WGCNA network construction; Figure S3: Hierarchical clustering of samples prior to WGCNA; Figure S4: Relationships among module eigengenes identified through WGCNA; Figure S5: Phylogenetic relationships of trypsin-like serine proteases from *Helicoverpa armigera* and related lepidopteran species; Figure S6: Phylogenetic relationships of chymotrypsin-like serine proteases from *Helicoverpa armigera* and *Helicoverpa zea*; Table S1: Functional annotation of genes within the midgut-associated turquoise co-expression module of *Helicoverpa armigera*.
