# Supplementary material for: RNA-seq Co-Expression Analysis Reveals a Midgut-Associated Digestive Gene Module in Helicoverpa armigera
Source: BioTech (Basel). 2026 Jul 13;15(3):53. doi: 10.3390/biotech15030053 (PMC13398162; doi:10.3390/biotech15030053)
Supplement: Supplementary file 1 [file biotech-15-00053-s001.zip › biotech-4365100-supplementary.pdf]

BioTech

Supplementary Materials: RNA-seq Co-Expression Analysis Reveals a Midgut-Associated Digestive Gene Module in Helicoverpa armigera

Bairon J. Matabanchoy Pejendino†, Vicente E. Mallama Cadena†, María C. Díaz Rodríguez, Claudia Salazar Gonzalez and Pedro A. Velasquez-Vasconez

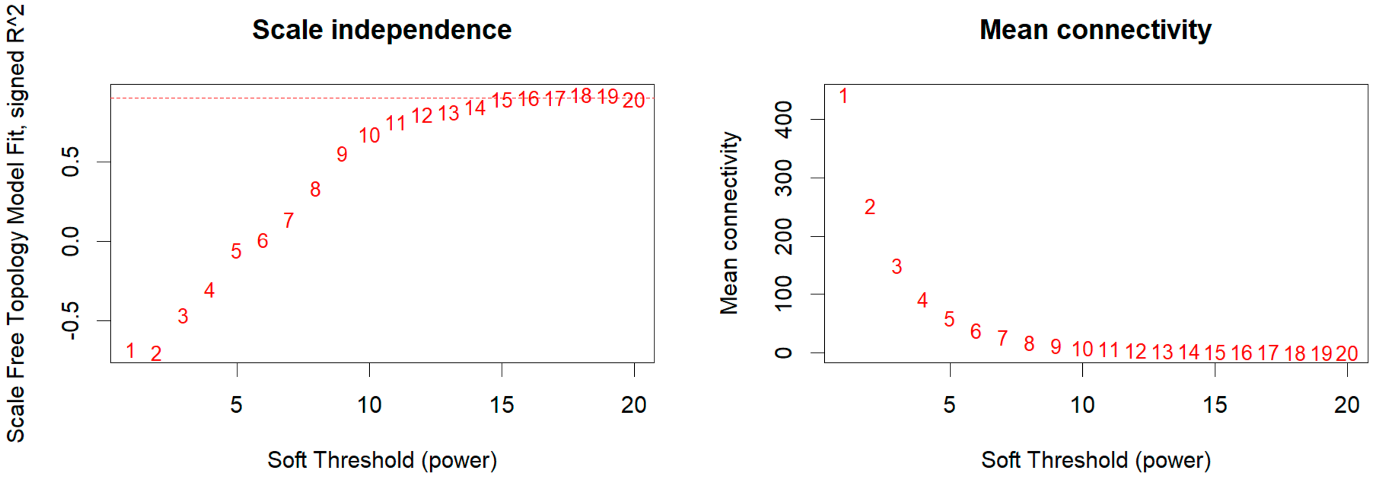

Figure S1. Selection of the soft-thresholding power for weighted gene co-expression network analysis (WGCNA). (A) Scale-free topology model fit index (signed R^2) across candidate soft-thresholding powers, with the dashed horizontal line indicating the target threshold for scale-free topology fit. (B) Mean connectivity across candidate soft-thresholding powers, showing the expected decrease in network connectivity as the weighting criterion becomes more stringent.

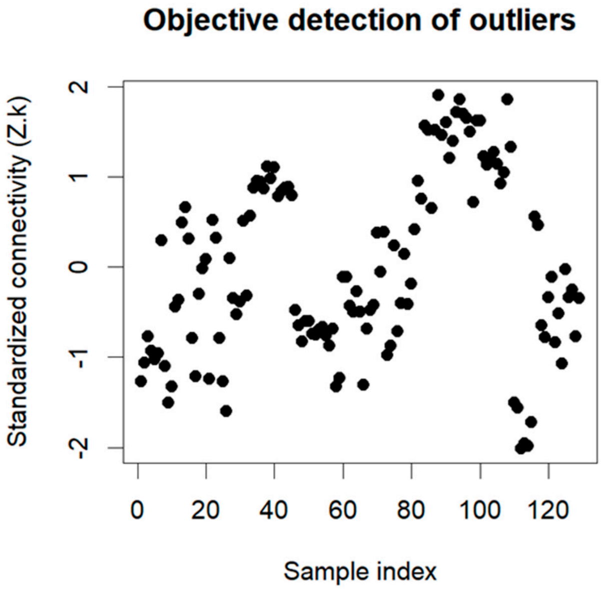

Figure S2. Objective detection of outlier samples prior to WGCNA network construction. Standardized sample connectivity (Z.k) was calculated for each sample to identify potentially divergent expression profiles. Connectivity values ranged approximately from −2.0 to 1.9, with no samples showing markedly reduced connectivity indicative of strong outlier behaviour.

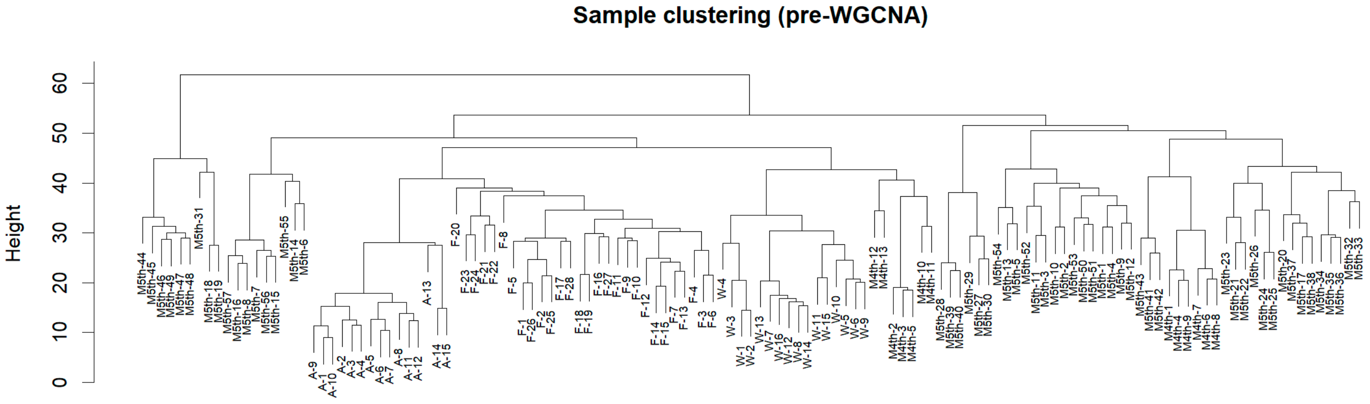

Figure S3. Hierarchical clustering of samples prior to weighted gene co-expression network analysis (WGCNA). The dendrogram shows that samples generally tended to group according to their biological source, including adult antennae (A), pupal fat body (F), whole-body early-instar samples (W), fourth-instar midgut (M4th) and fifth-instar midgut (M5th), although some heterogeneity was observed within tissue groups.

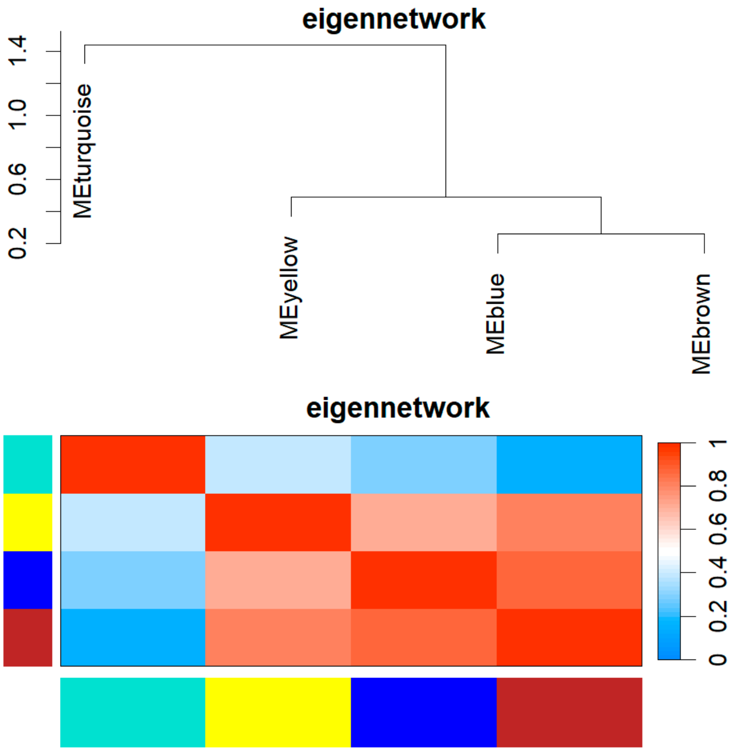

Figure S4. Relationships among module eigengenes identified through weighted gene co-expression network analysis (WGCNA). The upper panel shows the hierarchical clustering dendrogram of module eigengenes based on eigengene dissimilarity, whereas the lower panel presents the corresponding eigengene adjacency heatmap. Warmer colours indicate greater similarity between module eigengenes, while cooler colours indicate lower similarity.

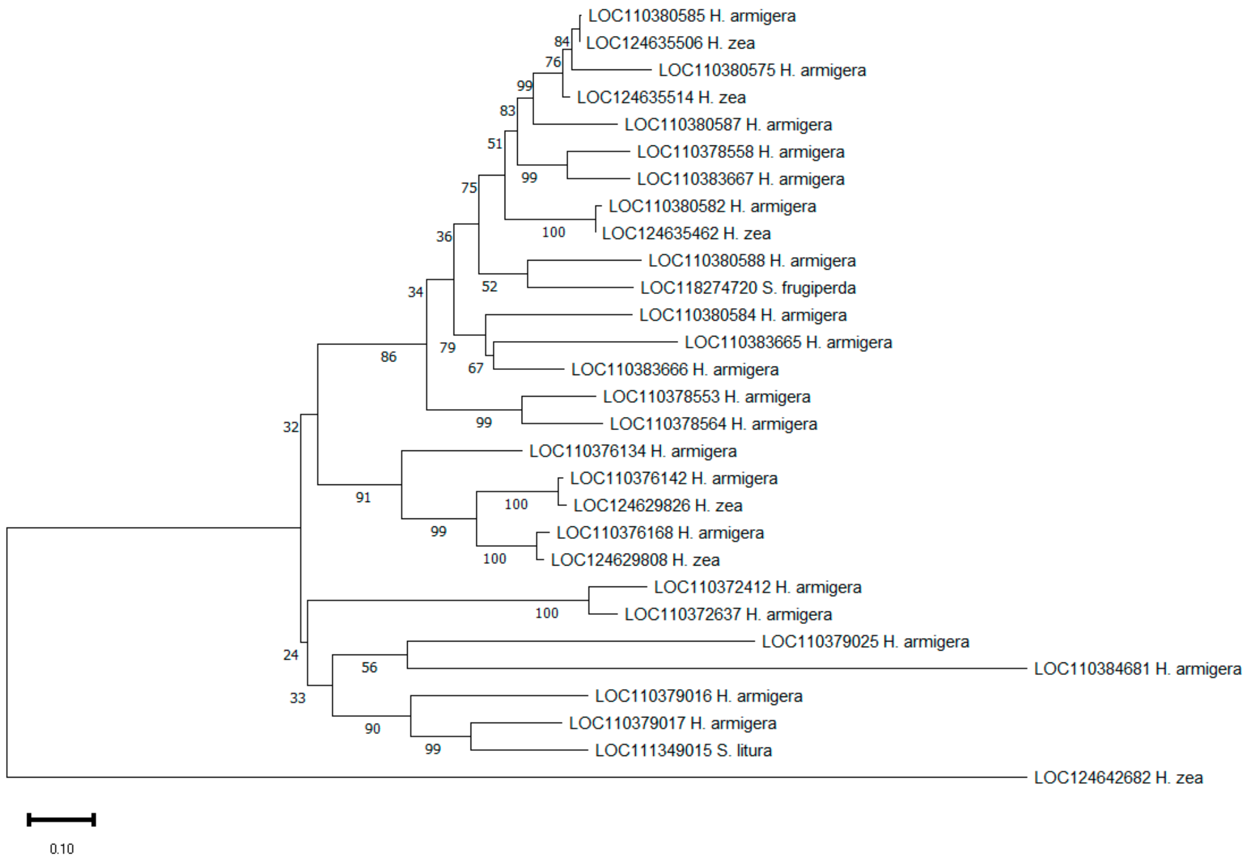

Figure S5. Phylogenetic relationships of trypsin-like serine proteases from *Helicoverpa armigera* and related lepidopteran species.

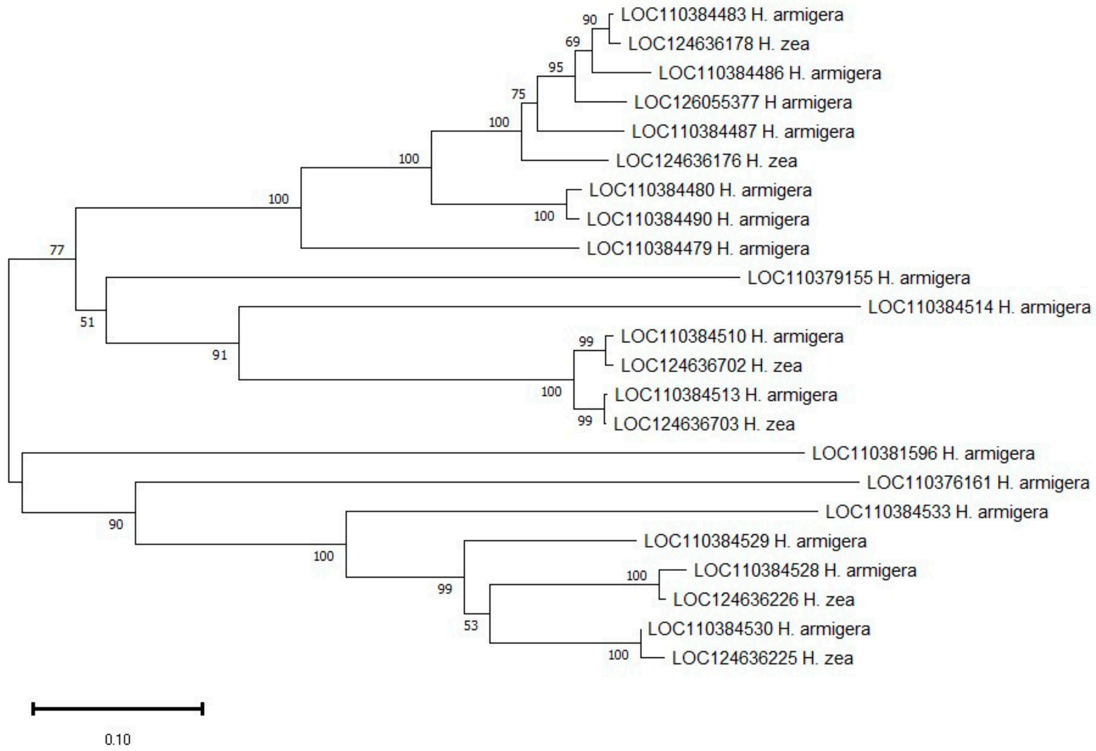

Figure S6. Phylogenetic relationships of chymotrypsin-like serine proteases from *Helicoverpa armigera* and *Helicoverpa zea*

Table S1. Functional annotation of genes within the midgut-associated turquoise co-expression module of *Helicoverpa armigera.*

Locus_ID	Module (WGCNA)	Encoded_product
LOC110369679	turquoise	small integral membrane protein 14
LOC110369917	turquoise	inositol-trisphosphate 3-kinase homolog, transcript variant X2; inositol-trisphosphate 3-kinase homolog, transcript variant X1; inositol-trisphosphate 3-kinase homolog, transcript variant X5; inositol-trisphosphate 3-kinase homolog, transcript variant X3; inositol-trisphosphate 3-kinase homolog, transcript variant X4
LOC110369929	turquoise	hyccin
LOC110370021	turquoise	probable serine hydrolase
LOC110370199	turquoise	leukocyte receptor cluster member 8
LOC110370218	turquoise	multiple C2 and transmembrane domain-containing protein
LOC110370292	turquoise	organic cation transporter protein, transcript variant X1; organic cation transporter protein, transcript variant X2
LOC110370385	turquoise	arrestin domain-containing protein 2, transcript variant X2; arrestin domain-containing protein 2, transcript variant X1
LOC110370405	turquoise	uncharacterized LOC110370405
LOC110370419	turquoise	soluble scavenger receptor cysteine-rich domain-containing protein SSC5D
LOC110370541	turquoise	calcium and integrin-binding protein 1
LOC110370611	turquoise	carboxylic ester hydrolase
LOC110370896	turquoise	glucose dehydrogenase [FAD, quinone], transcript variant X1; glucose dehydrogenase [FAD, quinone], transcript variant X2
LOC110370999	turquoise	proton-coupled amino acid transporter-like protein pathetic, transcript variant X2; proton-coupled amino acid transporter-like protein pathetic, transcript variant X1
LOC110371058	turquoise	myosin-VIIa, transcript variant X1; myosin-VIIa, transcript variant X2
LOC110371248	turquoise	centromere protein X
LOC110371255	turquoise	protein PRRC1
LOC110371306	turquoise	(2R)-3-sulfolactate dehydrogenase (NADP(+)), transcript variant X3; (2R)-3-sulfolactate dehydrogenase (NADP(+)), transcript variant X1; (2R)-3-sulfolactate dehydrogenase (NADP(+)), transcript variant X2
LOC110371319	turquoise	growth factor receptor-bound protein 2
LOC110371395	turquoise	uncharacterized LOC110371395
LOC110371406	turquoise	putative inorganic phosphate cotransporter
LOC110371411	turquoise	myrosinase 1
LOC110371435	turquoise	uncharacterized LOC110371435
LOC110371516	turquoise	protein Tob1, transcript variant X1; protein Tob1, transcript variant X2
LOC110371530	turquoise	MFS-type transporter SLC18B1
LOC110371578	turquoise	uncharacterized LOC110371578
LOC110371751	turquoise	cytochrome P450 6B5
LOC110371805	turquoise	uncharacterized LOC110371805
LOC110371807	turquoise	facilitated trehalose transporter Tret1
LOC110371810	turquoise	uncharacterized LOC110371810
LOC110371836	turquoise	uncharacterized LOC110371836, transcript variant X1; uncharacterized LOC110371836, transcript variant X2
LOC110372031	turquoise	uncharacterized LOC110372031
LOC110372045	turquoise	adenylosuccinate lyase
LOC110372049	turquoise	sulfotransferase 1E1
LOC110372057	turquoise	alkyldihydroxyacetonephosphate synthase
LOC110372081	turquoise	carboxypeptidase N subunit 2
LOC110372217	turquoise	myoblast growth factor receptor egl-15, transcript variant X1; myoblast growth factor receptor egl-15, transcript variant X2
LOC110372234	turquoise	translocation protein SEC62, transcript variant X1; translocation protein SEC62, transcript variant X2
LOC110372273	turquoise	branched-chain-amino-acid aminotransferase, cytosolic
LOC110372311	turquoise	uncharacterized LOC110372311
LOC110372322	turquoise	CCR4-NOT transcription complex subunit 7
LOC110372333	turquoise	organic cation transporter protein

LOC110372395	turquoise	growth/differentiation factor 8
LOC110372608	turquoise	luciferin sulfotransferase
LOC110372794	turquoise	uncharacterized LOC110372794
LOC110372811	turquoise	lysosomal-associated transmembrane protein 4A, transcript variant X2; lysosomal-associated transmembrane protein 4A, transcript variant X3; lysosomal-associated transmembrane protein 4A, transcript variant X1
LOC110372914	turquoise	uncharacterized LOC110372914
LOC110373019	turquoise	carcinine transporter
LOC110373076	turquoise	eukaryotic translation initiation factor 4E-binding protein Mextli, transcript variant X4; eukaryotic translation initiation factor 4E-binding protein Mextli, transcript variant X3; eukaryotic translation initiation factor 4E-binding protein Mextli, transcript variant X8; eukaryotic translation initiation factor 4E-binding protein Mextli, transcript variant X9; eukaryotic translation initiation factor 4E-binding protein Mextli, transcript variant X5; eukaryotic translation initiation factor 4E-binding protein Mextli, transcript variant X2; eukaryotic translation initiation factor 4E-binding protein Mextli, transcript variant X7; eukaryotic translation initiation factor 4E-binding protein Mextli, transcript variant X1; eukaryotic translation initiation factor 4E-binding protein Mextli, transcript variant X6; eukaryotic translation initiation factor 4E-binding protein Mextli, transcript variant X10
LOC110373246	turquoise	glyoxylate reductase/hydroxypyruvate reductase
LOC110373322	turquoise	beta-1,4-N-acetylgalactosaminyltransferase bre-4, transcript variant X2; beta-1,4-N-acetylgalactosaminyltransferase bre-4, transcript variant X1
LOC110373329	turquoise	diuretic hormone class 2, transcript variant X1; diuretic hormone class 2, transcript variant X2
LOC110373330	turquoise	nose resistant to fluoxetine protein 6
LOC110373486	turquoise	putative defense protein 3
LOC110373545	turquoise	carboxypeptidase B
LOC110373579	turquoise	uncharacterized LOC110373579
LOC110373639	turquoise	vacuolar protein sorting-associated protein 37C
LOC110373931	turquoise	cytokine-like nuclear factor N-PAC, transcript variant X2; cytokine-like nuclear factor N-PAC, transcript variant X1; cytokine-like nuclear factor N-PAC, transcript variant X3
LOC110374055	turquoise	tachykinins, transcript variant X3; tachykinins, transcript variant X1; tachykinins, transcript variant X2
LOC110374084	turquoise	uncharacterized LOC110374084
LOC110374137	turquoise	uncharacterized LOC110374137
LOC110374462	turquoise	mediator of RNA polymerase II transcription subunit 22
LOC110374717	turquoise	uncharacterized LOC110374717
LOC110374797	turquoise	allatostatin
LOC110374799	turquoise	uncharacterized LOC110374799, transcript variant X1; uncharacterized LOC110374799, transcript variant X2
LOC110374824	turquoise	protein FAM32A-like
LOC110374834	turquoise	GATA-binding factor C, transcript variant X2; GATA-binding factor C, transcript variant X1; GATA-binding factor C, transcript variant X3; GATA-binding factor C, transcript variant X4; GATA-binding factor C, transcript variant X5
LOC110374859	turquoise	carbonyl reductase [NADPH] 1
LOC110374861	turquoise	uncharacterized LOC110374861, transcript variant X2; uncharacterized LOC110374861, transcript variant X1
LOC110374863	turquoise	myeloid differentiation primary response protein MyD88
LOC110374885	turquoise	mucin-17, transcript variant X2; mucin-17, transcript variant X3; mucin-17, transcript variant X4; mucin-17, transcript variant X5; mucin-17, transcript variant X1
LOC110374921	turquoise	uncharacterized LOC110374921
LOC110375075	turquoise	uncharacterized LOC110375075
LOC110375077	turquoise	homeotic protein labial
LOC110375154	turquoise	prion-like-(Q/N-rich) domain-bearing protein 96
LOC110375330	turquoise	ankyrin repeat domain-containing protein 17

LOC110375373	turquoise	histone-lysine N-methyltransferase PRDM16, transcript variant X1; histone-lysine N-methyltransferase PRDM16, transcript variant X2; histone-lysine N-methyltransferase PRDM16, transcript variant X5; histone-lysine N-methyltransferase PRDM16, transcript variant X6; histone-lysine N-methyltransferase PRDM16, transcript variant X3; histone-lysine N-methyltransferase PRDM16, transcript variant X4
LOC110375471	turquoise	uncharacterized LOC110375471
LOC110375478	turquoise	uncharacterized LOC110375478
LOC110375604	turquoise	fatty acid-binding protein
LOC110375627	turquoise	neo-calmodulin, transcript variant X2; neo-calmodulin, transcript variant X1
LOC110375628	turquoise	adenosine deaminase 2
LOC110375658	turquoise	chymotrypsin-2
LOC110375844	turquoise	acetylcholinesterase
LOC110375848	turquoise	prostaglandin reductase 1
LOC110375859	turquoise	prostaglandin reductase 1
LOC110375876	turquoise	myophilin
LOC110375907	turquoise	transducin beta-like protein 2, transcript variant X2; transducin beta-like protein 2, transcript variant X1
LOC110375993	turquoise	phospholipid-transporting ATPase ABCA3
LOC110376142	turquoise	trypsin, alkaline B
LOC110376185	turquoise	uncharacterized LOC110376185
LOC110376447	turquoise	lopap
LOC110376475	turquoise	G protein-activated inward rectifier potassium channel 3, transcript variant X1; G protein-activated inward rectifier potassium channel 3, transcript variant X2; G protein-activated inward rectifier potassium channel 3, transcript variant X3; G protein-activated inward rectifier potassium channel 3, transcript variant X4; G protein-activated inward rectifier potassium channel 3, transcript variant X8; G protein-activated inward rectifier potassium channel 3, transcript variant X5; G protein-activated inward rectifier potassium channel 3, transcript variant X6; G protein-activated inward rectifier potassium channel 3, transcript variant X7
LOC110376497	turquoise	transmembrane reductase CYB561D2
LOC110376519	turquoise	serine hydroxymethyltransferase, transcript variant X3; serine hydroxymethyltransferase, transcript variant X1; serine hydroxymethyltransferase, transcript variant X7; serine hydroxymethyltransferase, transcript variant X4; serine hydroxymethyltransferase, transcript variant X5; serine hydroxymethyltransferase, transcript variant X2; serine hydroxymethyltransferase, transcript variant X6
LOC110376528	turquoise	lactosylceramide 4-alpha-galactosyltransferase
LOC110376784	turquoise	solute carrier organic anion transporter family member 2A1
LOC110376854	turquoise	DNA-directed RNA polymerases I, II, and III subunit RPABC2
LOC110376952	turquoise	zinc transporter ZIP10
LOC110377051	turquoise	collagenase
LOC110377110	turquoise	facilitated trehalose transporter Tret1
LOC110377156	turquoise	glutathione S-transferase 1
LOC110377183	turquoise	4-galactosyl-N-acetylglucosaminide 3-alpha-L-fucosyltransferase 9
LOC110377186	turquoise	serine-threonine kinase receptor-associated protein
LOC110377314	turquoise	uncharacterized LOC110377314, transcript variant X2; uncharacterized LOC110377314, transcript variant X1
LOC110377322	turquoise	homeobox protein CDX-1, transcript variant X1; homeobox protein CDX-1, transcript variant X2; homeobox protein CDX-1, transcript variant X3
LOC110377358	turquoise	carboxylesterase 3A
LOC110377403	turquoise	juvenile hormone esterase
LOC110377439	turquoise	ATP-sensitive inward rectifier potassium channel 12
LOC110377469	turquoise	unc-112-related protein
LOC110377483	turquoise	juvenile hormone-binding protein
LOC110377689	turquoise	6-phosphofructo-2-kinase/fructose-2,6-bisphosphatase
LOC110377790	turquoise	trans-1,2-dihydrobenzene-1,2-diol dehydrogenase

LOC110377849	turquoise	putative fatty acyl-CoA reductase CG5065, transcript variant X2; putative fatty acyl-CoA reductase CG5065, transcript variant X1
LOC110377890	turquoise	organic cation transporter protein
LOC110377891	turquoise	organic cation transporter protein
LOC110378095	turquoise	probable ATP-dependent RNA helicase DDX46
LOC110378149	turquoise	zinc-type alcohol dehydrogenase-like protein SERP1785
LOC110378206	turquoise	pleckstrin homology domain-containing family G member 5, transcript variant X9; pleckstrin homology domain-containing family G member 5, transcript variant X2; pleckstrin homology domain-containing family G member 5, transcript variant X1; pleckstrin homology domain-containing family G member 5, transcript variant X6; pleckstrin homology domain-containing family G member 5, transcript variant X4; pleckstrin homology domain-containing family G member 5, transcript variant X8; pleckstrin homology domain-containing family G member 5, transcript variant X3; pleckstrin homology domain-containing family G member 5, transcript variant X5; pleckstrin homology domain-containing family G member 5, transcript variant X7; pleckstrin homology domain-containing family G member 5, transcript variant X10
LOC110378372	turquoise	enoyl-CoA delta isomerase 1, mitochondrial, transcript variant X2; enoyl-CoA delta isomerase 1, mitochondrial, transcript variant X1
LOC110378478	turquoise	15-hydroxyprostaglandin dehydrogenase [NAD(+)]
LOC110378480	turquoise	alcohol dehydrogenase 2
LOC110378510	turquoise	slit homolog 1 protein
LOC110378529	turquoise	zinc-type alcohol dehydrogenase-like protein SERP1785
LOC110378553	turquoise	trypsin, alkaline B
LOC110378554	turquoise	uncharacterized LOC110378554
LOC110378939	turquoise	uncharacterized LOC110378939
LOC110378961	turquoise	zinc finger protein 37, transcript variant X1; zinc finger protein 37, transcript variant X3; zinc finger protein 37, transcript variant X6; zinc finger protein 37, transcript variant X5; zinc finger protein 37, transcript variant X2; zinc finger protein 37, transcript variant X4
LOC110379413	turquoise	coiled-coil domain-containing protein 25
LOC110379590	turquoise	membrane-bound alkaline phosphatase
LOC110379719	turquoise	putative inorganic phosphate cotransporter
LOC110379816	turquoise	serine/arginine repetitive matrix protein 3, transcript variant X1; serine/arginine repetitive matrix protein 3, transcript variant X5; serine/arginine repetitive matrix protein 3, transcript variant X2; serine/arginine repetitive matrix protein 3, transcript variant X4; serine/arginine repetitive matrix protein 3, transcript variant X3
LOC110379817	turquoise	POU domain protein 2, transcript variant X9; POU domain protein 2, transcript variant X2; POU domain protein 2, transcript variant X8; POU domain protein 2, transcript variant X3; POU domain protein 2, transcript variant X6; POU domain protein 2, transcript variant X1; POU domain protein 2, transcript variant X7; POU domain protein 2, transcript variant X5; POU domain protein 2, transcript variant X4
LOC110379848	turquoise	uncharacterized LOC110379848
LOC110380023	turquoise	uncharacterized LOC110380023
LOC110380050	turquoise	peroxisomal membrane protein 11C
LOC110380162	turquoise	homeobox protein OTX1, transcript variant X1; homeobox protein OTX1, transcript variant X2
LOC110380174	turquoise	high affinity cationic amino acid transporter 1, transcript variant X1; high affinity cationic amino acid transporter 1, transcript variant X2
LOC110380202	turquoise	uncharacterized LOC110380202, transcript variant X2; uncharacterized LOC110380202, transcript variant X1
LOC110380203	turquoise	luciferin 4-monooxygenase
LOC110380223	turquoise	DNA-binding protein RFXANK
LOC110380230	turquoise	uncharacterized LOC110380230
LOC110380291	turquoise	5-hydroxytryptamine receptor 1, transcript variant X1; 5-hydroxytryptamine receptor 1, transcript variant X2
LOC110380471	turquoise	ras-related GTP-binding protein C
LOC110380603	turquoise	sodium/potassium-transporting ATPase subunit beta-2

LOC110380607	turquoise	uncharacterized LOC110380607, transcript variant X1; uncharacterized LOC110380607, transcript variant X2
LOC110380624	turquoise	myotubularin-related protein 4, transcript variant X6; myotubularin-related protein 4, transcript variant X1; myotubularin-related protein 4, transcript variant X2; myotubularin-related protein 4, transcript variant X3; myotubularin-related protein 4, transcript variant X4; myotubularin-related protein 4, transcript variant X5
LOC110380846	turquoise	myrosinase 1
LOC110380941	turquoise	bile salt-activated lipase
LOC110381163	turquoise	carbonyl reductase [NADPH] 1
LOC110381164	turquoise	lactase/phlorizin hydrolase
LOC110381174	turquoise	gastrula zinc finger protein XlCGF17.1
LOC110381256	turquoise	ATP synthase subunit s, mitochondrial
LOC110381279	turquoise	venom carboxylesterase-6
LOC110381495	turquoise	macro domain-containing protein PG1779
LOC110381546	turquoise	splicing factor 1, transcript variant X4; splicing factor 1, transcript variant X2; splicing factor 1, transcript variant X5; splicing factor 1, transcript variant X1; splicing factor 1, transcript variant X3
LOC110381605	turquoise	peroxisomal membrane protein PEX13
LOC110381632	turquoise	uncharacterized LOC110381632, transcript variant X1; uncharacterized LOC110381632, transcript variant X2
LOC110381726	turquoise	venom serine protease 34, transcript variant X1; venom serine protease 34, transcript variant X3; venom serine protease 34, transcript variant X2
LOC110381752	turquoise	peptidyl-prolyl cis-trans isomerase Fkbp12
LOC110382087	turquoise	uncharacterized LOC110382087, transcript variant X1; uncharacterized LOC110382087, transcript variant X2
LOC110382241	turquoise	integrin beta-nu
LOC110382308	turquoise	neuropeptide CCHamide-2, transcript variant X1; neuropeptide CCHamide-2, transcript variant X3; neuropeptide CCHamide-2, transcript variant X2; neuropeptide CCHamide-2, transcript variant X4
LOC110382319	turquoise	uncharacterized LOC110382319
LOC110382762	turquoise	UDP-glycosyltransferase UGT5
LOC110382776	turquoise	putative fatty acyl-CoA reductase CG5065, transcript variant X1; putative fatty acyl-CoA reductase CG5065, transcript variant X3; putative fatty acyl-CoA reductase CG5065, transcript variant X2
LOC110382874	turquoise	thioredoxin domain-containing protein 9
LOC110382923	turquoise	SWI/SNF-related matrix-associated actin-dependent regulator of chromatin subfamily E member 1, transcript variant X2; SWI/SNF-related matrix-associated actin-dependent regulator of chromatin subfamily E member 1, transcript variant X1; SWI/SNF-related matrix-associated actin-dependent regulator of chromatin subfamily E member 1, transcript variant X4; SWI/SNF-related matrix-associated actin-dependent regulator of chromatin subfamily E member 1, transcript variant X3; SWI/SNF-related matrix-associated actin-dependent regulator of chromatin subfamily E member 1, transcript variant X5
LOC110382948	turquoise	uncharacterized LOC110382948, transcript variant X1; uncharacterized LOC110382948, transcript variant X2
LOC110383179	turquoise	pancreatic lipase-related protein 2, transcript variant X1; pancreatic lipase-related protein 2, transcript variant X2
LOC110383249	turquoise	facilitated trehalose transporter Tret1
LOC110383424	turquoise	S-adenosylmethionine decarboxylase proenzyme, transcript variant X1; S-adenosylmethionine decarboxylase proenzyme, transcript variant X2
LOC110383619	turquoise	fork head domain transcription factor slp2
LOC110383815	turquoise	protein bunched, class 2/F/G isoform, transcript variant X1; protein bunched, class 2/F/G isoform, transcript variant X3; protein bunched, class 2/F/G isoform, transcript variant X2
LOC110383829	turquoise	adenylyl cyclase X E, transcript variant X3; adenylyl cyclase X E, transcript variant X1; adenylyl cyclase X E, transcript variant X2

LOC110383871	turquoise	low-density lipoprotein receptor, transcript variant X4; low-density lipoprotein receptor, transcript variant X3; low-density lipoprotein receptor, transcript variant X7; low-density lipoprotein receptor, transcript variant X5; low-density lipoprotein receptor, transcript variant X1; low-density lipoprotein receptor, transcript variant X6; low-density lipoprotein receptor, transcript variant X2; low-density lipoprotein receptor, transcript variant X11; low-density lipoprotein receptor, transcript variant X12; low-density lipoprotein receptor, transcript variant X8; low-density lipoprotein receptor, transcript variant X9; low-density lipoprotein receptor, transcript variant X10
LOC110383911	turquoise	uncharacterized LOC110383911, transcript variant X2; uncharacterized LOC110383911, transcript variant X1; uncharacterized LOC110383911, transcript variant X4; uncharacterized LOC110383911, transcript variant X5; uncharacterized LOC110383911, transcript variant X3
LOC110383936	turquoise	major facilitator superfamily domain-containing protein 9-like
LOC110384036	turquoise	aldehyde dehydrogenase 1A1
LOC110384045	turquoise	trypsin-3
LOC110384279	turquoise	mortality factor 4-like protein 1
LOC110384294	turquoise	survival motor neuron protein
LOC110384341	turquoise	general transcription factor IIE subunit 2
LOC110384359	turquoise	anaphase-promoting complex subunit 11, transcript variant X1; anaphase-promoting complex subunit 11, transcript variant X2; anaphase-promoting complex subunit 11, transcript variant X3
LOC110384398	turquoise	facilitated trehalose transporter Tret1
LOC110384410	turquoise	facilitated trehalose transporter Tret1-2 homolog
LOC110384445	turquoise	male-enhanced antigen 1
LOC126053463	turquoise	multidrug resistance protein homolog 49
LOC126054033	turquoise	uncharacterized LOC126054033
LOC126054285	turquoise	sodium-independent sulfate anion transporter-like
LOC126054553	turquoise	uncharacterized LOC126054553
LOC126054783	turquoise	carboxypeptidase B
LOC126055276	turquoise	15-hydroxyprostaglandin dehydrogenase [NAD(+)]-like
LOC126055386	turquoise	uncharacterized LOC126055386, transcript variant X3; uncharacterized LOC126055386, transcript variant X4; uncharacterized LOC126055386, transcript variant X2; uncharacterized LOC126055386, transcript variant X1
LOC126055498	turquoise	uncharacterized LOC126055498
LOC126055880	turquoise	uncharacterized LOC126055880, transcript variant X1; uncharacterized LOC126055880, transcript variant X2
LOC126056109	turquoise	uncharacterized LOC126056109
LOC126056210	turquoise	uncharacterized LOC126056210
LOC126056312	turquoise	high mobility group protein D
LOC126056509	turquoise	uncharacterized oxidoreductase dhs-27
LOC126056532	turquoise	UDP-glycosyltransferase UGT5
LOC126056931	turquoise	uncharacterized LOC126056931
